# Efficient and inexpensive transient expression of multispecific multivalent antibodies in Expi293 cells

**DOI:** 10.1186/s12575-017-0060-7

**Published:** 2017-09-15

**Authors:** Xiaotian T. Fang, Dag Sehlin, Lars Lannfelt, Stina Syvänen, Greta Hultqvist

**Affiliations:** 1 Department of Public Health and Caring Sciences, Uppsala University, Rudbeck laboratory, Dag Hammarskjöldsväg 20, 751 85 Uppsala, Sweden; 2 Department of Pharmaceutical biosciences, Uppsala University, Biomedical center, Husargatan 3, Box 591, 75124 Uppsala, Sweden

**Keywords:** Bispecific antibodies, Expi293, HEK293, PEI, Transient protein expression

## Abstract

**Background:**

Immunotherapy is a very fast expanding field within drug discovery and, hence, rapid and inexpensive expression of antibodies would be extremely valuable. Antibodies are, however, difficult to express. Multifunctional antibodies with additional binding domains further complicate the expression. Only few protocols describe the production of tetravalent bispecific antibodies and all with limited expression levels.﻿

**Methods:**

Here, we describe a protocol that can produce functional tetravalent, bispecific antibodies at around 22 mg protein/l to a low cost. The expression system is based on the Expi293 cells, which have been adapted to grow in denser cultures than HEK293 cells and gives higher expression yields. The new protocol transfects the E﻿xpi293 cells with PEI (which has a negligible cost).

**Results:**

The protocol has been used to generate multiple variants of tetra- and hexavalent bispecific antibodies with yields of around 22 mg protein/l within 10 days. All materials are commercially available and the implementation of the protocol is inexpensive and straightforward. The bispecific antibodies generated in our lab were capable of binding to all antigens with similar affinity as the original antibody. Two of the bispecific antibodies have also been used in transgenic mice as positron emission tomography (PET) ligands to successfully detect amyloid-beta (Aβ) aggregates *in vivo*.

**Conclusions:**

This protocol is the first describing transfection of the human Expi293 cells with PEI. It can be used to generate functional multi-specific antibodies in high amounts. The use of biological drugs, and in particular multispecific antibodies, is rapidly increasing, hence improved protocols such as the one presented here are highly valuable.

## Background

**Fig. 1:**
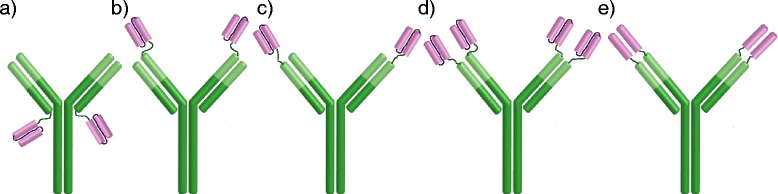
Different antibodies that have been produced according to the protocol presented in this paper. **a, b, c** Tetravalent bispecific antibodies where scFvs have been connected to an IgG at the heavy or light chain terminals. **d** Hexavalent bispecific antibody where scFvs have been connected to the N- terminal of both the heavy and light chains. **e** DVD antibody where the variable heavy domain and the variable light chain domain have been attached to the corresponding domains of the IgG

Immunotherapy is today a very fast advancing fields within drug discovery. Efforts are being made to develop antibodies that can be used to treat a large number of different diseases. With the recent advances in multifunctional antibody design the possibilities have increased even further. For efficient testing of potential drug candidates, it is essential to have a fast and inexpensive system to express many different antibodies. However, antibodies are difficult to expresses due to their large size. Furthermore, the two heavy and two light chains need to be paired correctly. Multispecific antibodies with additional binding domains, e.g. tetravalent bispecific antibodies (Fig. 1), further complicate the expression.

For many applications, post-translational modifications are essential and hence an expression system as similar to the human body as possible is desired. Traditionally, stable cell lines expressing antibodies were a necessity to generate sufficient amounts of the antibody, even for validation experiments. However, there has been a lot of progress in the last decade in transient expression of recombinant proteins. For instance, the robust and easily transfected HEK293 cell line has been adapted to growth in suspension [1]. Furthermore, it has been discovered that addition of cell cycle inhibiting substances after the transfection step will increase the protein yield [2, 3]. Promoters such as CMW and SV40 and other regulatory elements have been improved [4]. Alternative transfection techniques have been discovered and upgraded. It was shown that polyethylenimine (PEI) can be used to transfect HEK293 cells [5]. Most of the protocols for the production of recombinant proteins at high yields are based on the HEK293EBNA1-6E cell line that expresses a truncated version of the Epstein–Barr nuclear antigen 1 (EBNA1), which increases protein expression [6, 7]. However, this cell line is not commercially available anymore and patents restrict its commercial use.

**Fig. 2:**
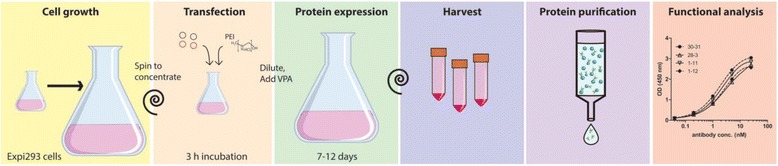
Schematic overview of the protocol

There are only a few protocols describing the purification of tetravalent bispecific antibodies and these methods have only achieved limited expression levels [8–10]. Here, we describe an expression protocol that uses only commercially available materials and in a 10-day process can produce functional tetravalent, bispecific antibodies at 22 mg protein per liter culture to a low cost. See Fig. 2 for a schematic overview.

The expression system is based on the Expi293 cells (LifeTechnologies), that have been adapted to grow in denser cultures than normal HEK293 cells (the adapted cells are healthy at 5 million cells/ml) and give a higher expression yield. LifeTechnologies recommends to transfect this cell line with ExpiFectamine (which is an expensive reagent), making it less suitable for testing of multiple antibodies. In this protocol, Expi293 cells are transfected instead with PEI (which costs 1/1000 as much as ExpiFectamine) to generate multiple variants of tetra- and hexavalent bispecific antibodies.

## Methods

### Materials to prepare

A 1 mg/ml linear 40 kDa PEI Max solution was prepared by dissolving 1 g of linear 40 kDa PEI Max powder (24765–1, Polysciences) in 1 L Milli-Q water, and stirred until completely dissolved into solution, followed by sterile-filtering through a 0.22 μm filter, then aliquoted and stored at −20 °C.

A 0.5 M VPA (2-propyl-pentanoic acid, sodium salt) (P4543, Sigma Aldrich) solution was prepared by adding VPA to water and then sterilizing it through a 0.22 μm filter into sterile 50 ml tubes. A tube for frequent use was stored at 4 °C while the other aliquots were stored at −20 °C.

### Methods

#### Cloning

The genes to be expressed were cloned into the pcDNA3.4 vector (ThermoFisher). In general, cloning was made by GeneArt, ThermoFisher. The pcDNA3.4 vector uses the full length human cytomegalovirus (CMV) immediate- early promoter for high level gene expression together with the woodchuck posttranscriptional regulatory element (WPRE) downstream of the cloning site to enhance transcript expression. The gene, cloned into an equivalent vector, should express equally well. The antibodies’ natural signal peptides, located at the N-terminus, ensured that the antibodies were secreted to the cell medium. During the translocation, the signal peptide was cleaved off [11]. When expressing proteins that do not naturally have a signal peptide, a signal peptide from an antibody was added to induce secretion [12]. The two antibody chains were cloned into two different vectors. Codon usage was optimized for mammalian expression systems with GeneArt’s codon optimizer and their suggested Kozak sequence was used.

#### Amplification and purification of plasmid DNA

The cloned plasmid DNA was transfected to TOP10 cells (ThermoFisher) according to the instructions provided with the cells. Various kits were used to purify plasmid DNA, for instance GeneJET endo-free plasmid maxiprep (ThermoFisher), GeneJET Plasmid maxiprep (ThermoFisher) and QIAGENs maxiprep kit. The transfected bacteria were amplified in the recommended liquid medium (with ampicillin) and the plasmid purified according to the instructions for the plasmid purification kit used. The concentration was measured on a microvolume spectrophotometer (DeNovix DS-11). The DNA preparation should have an absorbance A_260_/A_280_ ratio of >1.8 to be considered clean enough for transfection.

#### Expansion of Expi293 cells

Expi293 cells (A14527, ThermoFisher) were used to express the multivalent antibodies. The recommendations for how to maintain and expand Expi293 cells were followed [13]. In short, the Expi293 cells were grown in Expi293 medium (A1435101, ThermoFisher) at 37°C, 125 rpm, 8% CO_2_ atmosphere (5% was also acceptable) with 80% humidity in plastic flasks with ventilated caps (Corning® Erlenmeyer sterile polycarbonate with 0.2 μm ventilated caps). A Minitron™ CO_2_ orbital shaker with 25 mm orbitals (Infors HT, Switzerland) was used for the incubation. These conditions were maintained throughout the protocol. During the maintenance and expansion phase, the cells were split to 0.3 million viable cells (mvc)/ml when they reached a density of 3–5 mvc/ml. This required splitting roughly every 4th day. The volumes used could be scaled up as long as a suitable size of flask was used, 30 ml Expi293 medium in a 125 ml flask was routinely used. Cell density was determined with a Nexcelom Auto T4 cell counter.

#### The day before transfection

It is important that the cells used for transfection display a viability of at least 95%, and have not reached a density higher than 3–5 mvc/ml prior to transfection. To avoid inhibition of the transfection by substances secreted by the cells, the medium was replaced the day before transfection by spinning down the cells at 200 x g for 10 min at RT in 225 ml centrifuge tubes (525–0506, VWR) and then carefully decanting or pipetting away all cell media before re-suspending the cells in pre-warmed fresh Expi293 medium. If the volumes were large, all cells were resuspended in a volume smaller than 50 ml and diluted afterwards. After the split, resuspension and dilution the cell concentration should be around 1 mvc/ ml. Transfection of 1000 mvc requires around 600 mvc the day before transfection. The cells were incubated under standard conditions until the day after.

#### Transfection

The cells in the culture were counted. 1000 mvc will ultimately give 1 l of transfected cells. The cells were spun at 200 x g for 10 min at RT. The medium was removed by carefully decanting or by aspiration. To reduce shear forces during the transfection, Pluronic® F-68 (A1288.0100, VWR) is added to the transfection medium, which was made by addition of Pluronic® F-68 to the Expi293 medium for an end concentration of 0.1% Pluronic® F-68. The cells were resuspended in the transfection medium at a final concentration of 20 mvc/ml, i.e. if the total number of cells were 1000 mvc, the cells were diluted in 50 ml transfection medium in. A suitable flask size for this transfection was 125 ml. 1.25 mg of plasmid DNA per 1000 mvc was added to the culture (can be added to the resuspension flask during the spin to make this step more efficient). When expressing antibodies, which have the heavy and light chain expressed on different plasmids, the total amount of DNA added should still be 1.25 mg. 70% of the DNA for the light chain plasmid and 30% for the heavy chain plasmid were routinely used. When the DNA and cells had been mixed, 3.75 ml per 1000 mvc of 1 mg/ml of linear 40 kDa PEI Max (24765–1, Polysciences) solution was added (see materials for preparation instructions) and the cells were incubated under standard conditions for 3 h. After incubation, the cells were transferred to a larger flask and diluted with pre-warmed Expi293 medium to a concentration of 1 mvc/ml (i.e. if the total number of cells was 1000 mvc, 950 ml pre-warmed Expi293 media was added to the 50 ml transfection culture). 0.5 M VPA (P4543, Sigma Aldrich) (see materials for preparation instructions) was added to a final concentration of 3.5 mM (i.e. if the total number of cells was 1000 mvc in 1 l, then 7 ml of 0.5 M VPA was added). The transfected cells were incubated for 7–12 days at 37°C, 125 rpm, 8% CO_2_ atmosphere without handling them.

#### Analysis of viability after the transfection

The viability of the cells was analyzed in a cell counter or microscope the day after transfection. At this point the cells usually had a viability of 80–95%. The cell viability was regularly checked every few days.

#### Analysis of protein expression

To assess antibody expression and functionality, aliquots of the cell media containing transfected cells were collected at regular intervals to determine the optimal harvesting day. The collected aliquots were spun at 13000 x g for 5 min, and the supernatant was analyzed with indirect ELISA, as described in [14], using an antigen relevant for the expressed antibody.

#### Harvest of transfected cells

Seven to twelve days after transfection the cells were harvested by centrifugation at 2000 x g for 15 min, but when the volume was larger than 200 ml then the cells were spun for 1 h to facilitate the subsequent filtering process. The supernatant was then filtered through a 0.22 μm filter.

#### Purification

Multivalent antibodies with an Fc domain can rapidly and reliably be purified with a HiTrap Protein G HP 5 ml column (17–0405-01, GE Healthcare). The filtered supernatant was loaded on the protein G column using an ÄKTA start system (GE Healthcare) according to the protein G protocol, using PBS to equilibrate the column (binding buffer) and 0.7% acetic acid (elution buffer) to elute the antibodies. The elution was monitored by measuring absorbance at 280 nm. Proteins without Fc domain but with a His-tag can be purified on a 5 ml HisTag Excel column (17–3712-05, GE Healthcare Life Sciences), which tolerates the chelators present in the Expi293 medium that make the use of traditional Ni2+ columns unsuitable for purification.

#### Buffer exchange

After purification, the antibodies were in a non-standardized buffer and the exact composition of the buffer depended on when the antibody was eluted. The antibodies were routinely stored in PBS and hence directly after the elution the buffer was exchanged, either by dialysis or by desalting columns with an appropriate molecular weight cut-off. When using the desalting columns, the proteins were first concentrated (see below).

#### Concentration of the protein sample

The antibodies were concentrated to a concentration of 0.5–30 mg/ml with Amicon ultra centrifugal filters (Merck Millipore) with a 50 kDa molecular weight cut-off.

#### Analysis of the protein concentration

The protein concentration was measured with a microvolume spectrophotometer (DeNovix DS-11) using the A_280_ setting. Since the amino acid sequence of the protein was known, the exact extinction coefficient could be calculated using the Expasy protein parameters software (http://web.expasy.org/protparam/) [15].

#### Functional assays

The antibodies that have been produced bind to multiple antigens. The affinities for these antigens were tested with indirect ELISA as previously described [14]. Briefly, ELISA plates were coated with the relevant antigen followed by incubation of serially diluted antibody, which was then detected with an HRP-conjugated anti-mouse-IgG and TMB (3,3′,5,5′-Tetramethylbenzidine) substrate. All produced antibodies showed the expected binding to their antigens. Some of the produced antibodies have also successfully been used as radioligands in positron emission tomography (PET) to detect amyloid-beta (Aβ) aggregates in transgenic mice [16].

## Results

### Different formats of antibodies

**Fig. 3:**
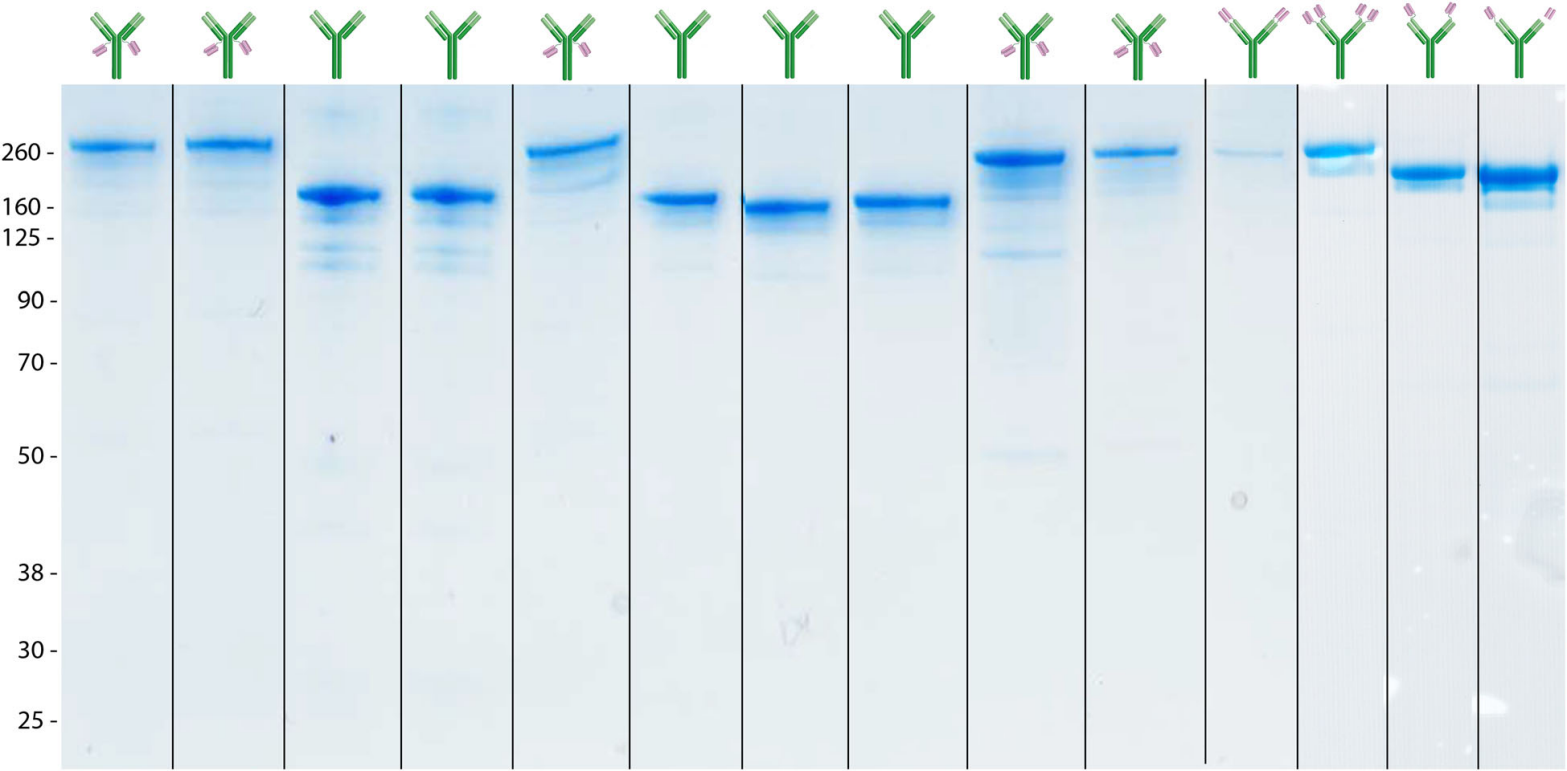
Non-reduced SDS-PAGE stained with Coomassie blue illustrating the purity of different antibody formats produced with suggested method and protein G purification. Schematic on top displays the type of antibody produced and its respective band on the gel. All samples are different antibodies but the same antibody format is represented multiple times. All antibodies were harvested 9–12 days post-transfection. Sample loaded on the gel were from after affinity purification

With the protocol presented here (Fig. 2), several different formats of multivalent antibodies have been expressed (Fig. 1) and the yields of some of these are presented in Table 1 and the purity is shown in Fig. 3. Some of the formats consist of single chain fragment variables (scFvs) attached to a standard IgG antibody (Fig. 1a-d) while some consist of a variable heavy or light domain attached to the variable heavy and light domains of an IgG respectively (Fig. 1e) (these are called DVD antibodies). No asymmetrical antibodies have been expressed with the protocol.

**Table 1:**
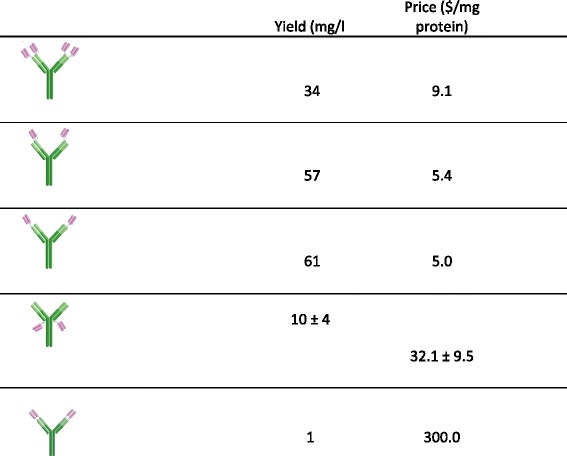
Yields obtained from different antibody formats with the protocol presented in this paper

The first three antibody variants (i.e. with one or two sdAbs attached to the N-terminus of the IgG) were expressed once

The fourth (i.e. with scFvs attached to the C-terminus of the light chain) was expressed 6 times and the mean ± SD of these experiment is stated

### Cell viability and antibody concentration post-transfection

**Fig. 4:**
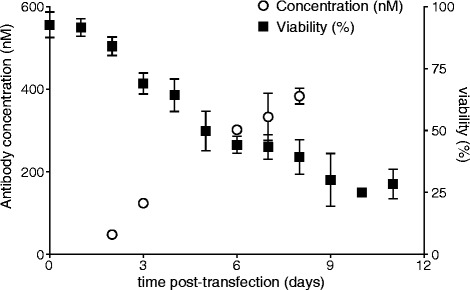
The efficiency of the transfection over time. Cell media when expressing different formats of antibodies 1 were sampled at several time points (2, 3, 6, 7, and 8 days post-transfection) and antibody concentration was measured by ELISA (*left* y-axis, *n* = 3 for day 6, 7, and 8 days post-transfection). *Right axis* is the cell viablity as measured in media samples obtained from several time points post-transfection (*n* = 5, *error bars* depict standard deviation)

Following transfection, the cell media was sampled in order to investigate the viability (%) of the cells, as well as the concentration of produced, functional antibody present in the media. Antibody concentration as measured by ELISA appeared to increase in a linear fashion up to the final measured time point (8 days). Cell viability decreases over time, reaching 25% on day 11 (Fig. 4).

### Purity and functionality of the expressed antibodies

**Fig. 5:**
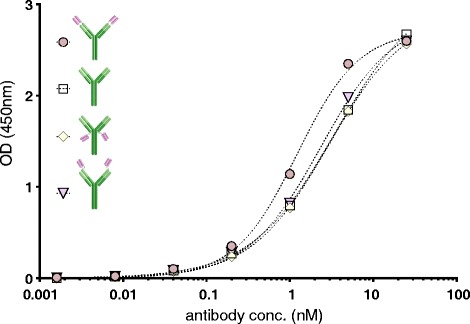
Affinity of the original antibody is retained even when additional binding sites are added. The affinity of the original antibody (in *green*) to its antigen was measured for a number of multivalent formats with ELISA

The purity of the protein purification was analysed by SDS-PAGE. Since the proteins were excreted and then affinity purified they were all pure (Fig. 3). Affinity of the expressed multivalent antibodies to their targets was analyzed with ELISA. All affinities were in the same range (K_d_ 2.5 ± 0.47 nM) as the original antibodies (Fig. 5). A scFv with affinity to the transferrin receptor (TfR) was attached to an antibody selectively binding to Aβ protofibrils in the format illustrated in Fig. 1a. The scFv binding to TfR enabled efficient transcytosis of the bispecific antibody across the blood-brain barrier, and hence, a radiolabeled version of the bispecific antibody can be used as a positron emission tomography (PET) ligand to visualize and quantify Aβ protofibrils in transgenic mice [16].

### Format expression efficiency

There were some differences in expression yields, but in general good yields were obtained for all studied antibody formats. The lowest efficiency was observed for the DVD antibody (Fig. 1e) which was obtained at a concentration of 1 mg/l of media. The yield of all the other antibody formats was 8 to 65 mg/l of media, with an average of around 22 mg/l of media.

### Comparison of expression efficiency and cost to with different transfection reagents

Companies that sell the different types of HEK293 cells usually promote variants of transfectamine as transfection reagents. Transfectamine and its variants are always expensive (approximately a thousand time more expensive than PEI). Transfection of Expi293 cells is routinely performed with ExpiFectamine transfection reagent. For expression of multiple variants of antibodies or large scale expression, the cost can easily become a limiting factor of the number/amount of antibody variants that can be produced and tested. Polyethylenimine (PEI) has proven to be an excellent replacement since it is inexpensive and can be used according to a simple protocol. It was first described by Boussif et al. [17]. The first PEI variant that was successfully used was linear PEI 25 kDa (Polysciences). This PEI is difficult to dissolve since it has to be dissolved in low pH but transfected at neutral pH. Instead, a more easily dissolved variant is used: PEI Max 40 kDa (Polysciences). The same expression levels were obtained with both PEI variants (data not shown), and since PEI max 40 kDa is easier to handle this variant was chosen for the experiments described in this study. The expression efficiency and cost per produced amount of antibody was compared with the PEI Max 40 kDa transfection protocol and the ExpiFectamine transfection protocol that ThermoFisher provides (Table 2).

**Table 2:** Bispecific antibody expression efficiency of the same construct

	Transfection reagent	Yield (mg/l)	Price ($/mg protein)	Costs ($/l media and transfection reagent)
	–			
	PEI	7	42	305
	–			
	ExpiFectamine	13	106	1380

The costs per transfected liter of media includes the cost of Expi293 media and PEI or ExpiFectamine

Here, results are shown from experiments performed at the same time to compare ExpiFectamine and PEI

## Discussion

Based on the cell viability and measured antibody concentration in the cell media following transfection, it appeared that the antibody should be harvested around 9–12 days post-transfection for optimal expression. Previously, other protocols have recommended to harvest after 7 days [13]. However, here antibody expression is observed to be ongoing on day 8 post-transfection (Fig. 4). Furthermore, the cell viability at this point has reached a fairly low percentage (25–35%). Therefore, while a true ‘optimal’ time of harvest cannot be recommended, a suitable point of harvest would be around 9–12 days post-transfection.

The published protocols of PEI transfection which result in similar yields as those reported in the present study have used HEK293E cells [5, 6, 18, 19]. The HEK293E line expresses an Epstein–Barr nuclear antigen 1 (EBNA1) which should increase the protein expression. The HEK293E cell line was previously sold by Invitrogen, but is no longer commercially available. Neither is it present in any of the cell line databases such as the European Collection of Authenticated Cell Cultures (ECACC) or the American Type Culture Collection (ATCC), and hence, there is an unmet need to find a commercially available cell line to use. A few years ago, the Expi293 cells were introduced on the market. The Expi293 cells can grow to higher cell densities than other HEK293 cells and therefore require less medium and less handling [20]. HEK293 cells require splitting every 2–3 days while the Expi293 cells only require splitting every 4–5 days, which also substantially reduces the amount of laboratory work required to maintain cell viability. The cell line also expresses proteins better than its predecessor [20]. The protocol described here is the first one that describes PEI transfection of Expi293 cells.

Up to 65 mg of multivalent antibody per liter of medium was obtained with the PEI transfection of Expi293 cells. ThermoFisher reported that only 56 out of 230 expressed proteins in Expi293 cells transfected with ExpiFectamine, were obtained in higher yields than 30 mg protein per liter and that 69 of 230 were expressed in yields of 1 mg protein per liter or less (https://www.thermofisher.com/se/en/home/life-science/protein-biology/protein-expression/mammalian-protein-expression/transient-mammalian-protein-expression/expi293-expression-system/expi293-protein-expression-data.html). Thus, the multivalent antibodies, which are complex proteins with multiple chains that need to be connected, were in the present study using PEI transfection obtained in similar yields as simpler proteins reported by ThermoFisher using ExpiFectamine.

Some of the previously published protocols that describe transfection of HEK293E cells with PEI include a step in which the cell medium is changed at transfection since it has been reported that some media may inhibit transfection [21]. For instance, Baldi et al. [18] suggested use of RPMI 1640 medium with Pluronic 68 during transfection. It has also been reported that media which inhibit PEI transfection do not do so if the cell density at transfection is high [22]. In the protocol, a cell concentration of 20 mvc/ml at the time of transfection is used, which is above the critical concentration according to Backliwal et al. Both RPMI 1640 and Expi293 media (both supplemented with 0.1% Pluronic 68) have been tested during transfection and slightly higher expression yields were obtained with Expi293 medium. Thus, the same medium can be used throughout the whole process, resulting in a simpler protocol than those requiring a switch of media at transfection.

There are only a few protocols describing transient expression of symmetric bispecific antibodies; all with lower yields than obtained with the present protocol. Orcutt et al. used HEK293 cells and PEI and obtained 5–7 mg protein per liter [9]. Pohl et al. used adherent cells which are more difficult to grow to large volumes with PEI and got 10 mg protein per liter [10]. Schanzer et al. used HEK293 cells and the expensive 293fectin (proprietary Thermofisher transfection reagent recommended for suspension 293 cells) and obtained 15–54 mg protein per liter [23]. They observed that an N- terminal fusion led to a 4–5 fold reduction in expression levels which was not observed in this study.

## Conclusions

The protocol outlined in this paper is the first describing transfection of the human Expi293 cells with PEI. It can be used to generate functional multi-specific antibodies in amounts comparable to those of other protocols that include considerably more expensive chemicals or are based on cells that are not available commercially. Since the use of biological drugs, and in particular multispecific antibodies, is rapidly increasing, improved protocols like the one presented here are of great value.

## Abbreviations

Aβ: Amyloid-beta
mvc: Million viable cells
PEI: Polyethylenimine
PET: Positron emission tomography
scFv: Single chain fragment variable
VPA: Valproic acid
